# 一个F13A1基因大片段缺失所致凝血因子XIII缺乏症家系的基因诊断

**DOI:** 10.3760/cma.j.issn.0253-2727.2023.01.011

**Published:** 2023-01

**Authors:** 亚玲 程, 子轩 丁, 丽娟 曹, 晶晶 韩, 健 苏, 歌阳 高, 自强 余, 霞 白, 兆钺 王, 长耿 阮

**Affiliations:** 1 苏州大学附属第一医院，江苏省血液研究所，国家血液系统疾病临床医学研究中心，国家卫生健康委员会血栓与止血重点实验室，血液学协同创新中心，苏州 215006 Collaborative Innovation Center of Hematology, National Clinical Research Center for Hematologic Diseases, NHC Key Laboratory of Thrombosis and Hemostasis, Jiangsu Institute of Hematology, the First Affiliated Hospital of Soochow University, Suzhou 215006, China; 2 苏州大学省部共建放射医学与辐射防护国家重点实验室，苏州 215123 State Key Laboratory of Radiation Medicine and Protection, Soochow University, Suzhou 215123, China

凝血因子XIII（FXIII）是凝血过程中的最后一个凝血因子，具有转谷氨酰胺酶活性，催化纤维蛋白单体中α和γ链中的谷氨酰胺酰胺基与赖氨酸氨基之间共价结合，从而形成不溶的纤维蛋白聚合物发挥止血作用[1]。血浆FXIII分别以2个A亚基（FXIII-A）和B亚基（FXIII-B）形成四聚体的形式存在，其中A亚基具有转谷氨酰胺酶活性，B亚基作为载体保护A亚基免于降解[2]–[3]。根据亚基缺陷类型FXIII缺乏症分为FXIII-A亚基缺乏症和FXIII-B亚基缺乏症两大类，后者临床出血表现较轻[4]–[5]。

遗传性FXIII-A缺乏症是一种罕见的常染色体隐性遗传的出血性疾病，发病率为1/200万～1/100万[6]–[7]。该类疾病的检测手段有限，常导致漏诊，二代测序（NGS）的普及大大改善了此类情况，但对基因大片段缺失的鉴定仍存在盲点。我们应用NGS技术对1个F13A1基因大片段缺失所致FXIII缺乏症病例及其家系成员进行鉴定和诊断，报道如下。

## 病例与方法

1. 家系资料：先证者，女，22岁，出生时脐带出血不止，轻微外伤后易发生肌肉、关节血肿，成年后发生2次黄体破裂出血，经血浆输注后好转。诊断明确后每4周输注200 ml血浆预防性治疗，患者未再发生出血事件并正常分娩一子。血常规：WBC 8.6×10^9^/L，RBC 3.98×10^12^/L，HGB 104 g/L，PLT 198×10^9^/L；活化部分凝血活酶时间（APTT）、凝血酶原时间（PT）、凝血酶时间（TT）、纤维蛋白原（Fbg）、抗凝血酶原Ⅲ活性（AT-AⅢ）、D-二聚体、纤维蛋白降解物（FDP）均在参考值范围内，肾功能正常。先证者父母及两兄长无出血表现，凝血指标正常。父母为近亲婚配。

2. 血标本的采集、处理和DNA提取：采集所有受试者外周血3 ml，0.109 mol/L枸橼酸钠（1∶9）抗凝，1 600×*g*离心15 min，分离血浆和血细胞；利用QIAamp DNA Blood Mini Kit（德国Qiagen公司产品）提取基因组DNA（按试剂商说明书操作），血浆与DNA置于−80 °C保存待用。

3. 尿素溶解法测定FXIII活性：受检血浆500 µl，加入0.025 mol/L氯化钙溶液500 µl，混合后置37 °C水浴中10～15 min，使凝块形成。将凝块移入5 mol/L尿素中，置于37 °C水浴中，观察凝块溶解时间。每5 min观察1次，2 h后每2～4 h观察1次，共观察24 h。

4. Western blot法检测血浆FXIII-A/B含量：取健康对照、先证者及其家系成员血浆标本，1 600×*g* 4 °C离心10 min，0.01 mol/L PBS 10倍稀释后，5×蛋白上样缓冲液混匀，99 °C加热10 min。在5％～10％梯度SDS-PAGE电泳分离目的蛋白，分离后的目的蛋白被转至硝酸纤维膜上，5％ BSA室温封闭1～2 h，重组Anti-Factor XIIIa和Anti-Factor XIIIb抗体（美国OriGene公司产品）1 000倍稀释，4 °C孵育过夜，洗膜后以HRP标记的鼠抗羊二抗室温孵育1～2 h，最后ECL显影。

5. NGS检测：利用本院的出血血栓疾病靶向基因测序组套对先证者和其父进行基因检测。将提取的DNA用超声波打断并制备DNA文库，通过捕获芯片对靶向基因的外显子及其临近的内含子区域的DNA进行富集，用通用引物对捕获的序列进行PCR扩增，在Ion S5测序平台对扩增产物进行NGS检测。最后使用IGV软件可视化分析NGS数据中的基因组变异情况。

6. PCR扩增及Sanger测序分析：根据F13A1基因组序列（NG_008107.1）和NGS检测原始数据设计两对引物（表1），目的片段覆盖基因F13A1的exon 12和部分intron 12，由上海生工生物工程公司合成。PCR体系（20 µl）：Premix Taq（TaKaRa Taq Version 2.0 plus dye）10 µl，DNA模板2 µl，正向引物（10 µmol/L）1 µl，反向引物（10 µmol/L）1 µl，ddH_2_O 6 µl。扩增条件：98 °C预变性5 min，95 °C变性30 s，60 °C退火30 s，72 °C延伸30 s；扩增35个循环后，72 °C延伸10 min。扩增结束后，取PCR产物进行琼脂糖凝胶电泳，双向Sanger测序。与NCBI所公布的基因F13A1的基因组DNA序列（NG_008107.1）进行核苷酸序列在线比对，以确定缺失范围和断裂位点。

**表1:** F13A1基因突变位置扩增引物序列

引物名称	正向引物序列（5′→3′）	反向引物序列（5′→3′）
F13A1-WT	TTGCCTGTCATTATCTCTGG	GACAGCGAGTCTCAGAAAG
F13A1-MT	AAGATAGACATTGCCAATTTTACCT	CAACAGTTTGAGCACAGAGGGTA

注 WT：野生型；MT：突变型

## 结果

1. 血浆FXIII活性检测结果：先证者的5 mol/L尿素溶解时间小于30 min，而正常对照及患者家系成员24 h不溶解，说明先证者血浆FXIII活性缺乏。

2. 血浆FXIII-A及FXIII-B抗原检测：Western blot结果显示先证者血浆FXIII-A蛋白条带缺失，其家系成员血浆FXIII-A蛋白条带与正常对照无差别（图1A、B）；而先证者血浆FXIII-B蛋白条带减弱，灰度扫描为正常人的73.6％，其家系成员与正常人无明显差别（图2A、B）。证实先证者血浆FXIII-A蛋白明显缺乏、FXIII-B蛋白轻度降低。

**图1:**
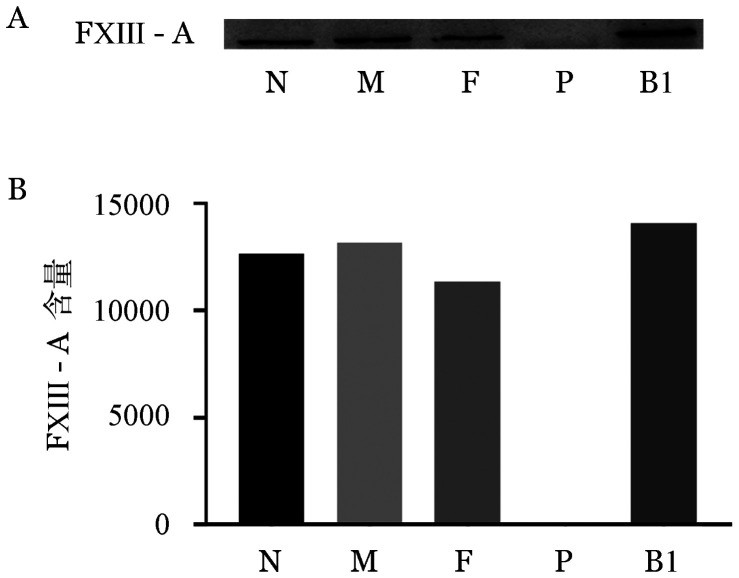
血浆FXIII-A抗原Western blot检测条带（A）和灰度扫描结果（B）（N为正常对照，F为患者父亲，M为患者母亲，P为患者，B1为患者兄长1）

**图2:**
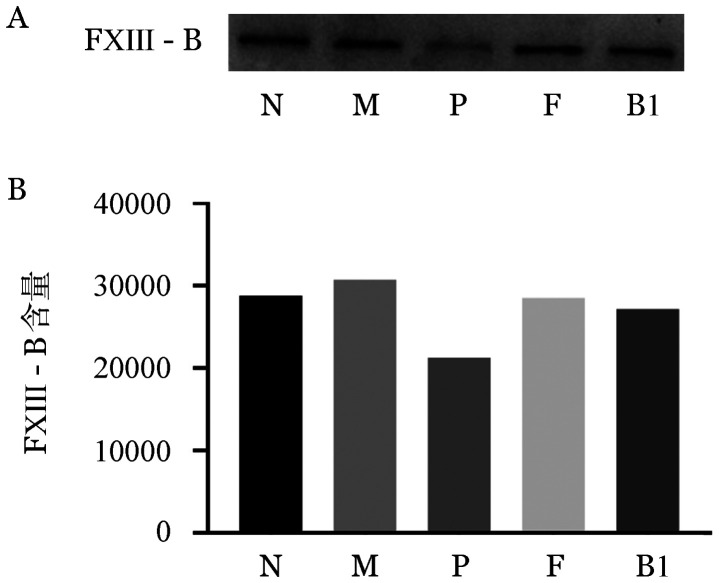
血浆FXIII-B抗原Western blot检测条带（A）和灰度扫描结果（B）（N为正常对照，F为父亲，M为母亲，P为先证者，B1为患者兄长1）

3. NGS技术检测F13A1基因突变：IGV软件分析显示先证者F13A1基因（NG_008107.1）exon 12信号全部缺失，exon 13正常（图3A）。其父该基因exon 12在g.150971位点出现信号断裂，断裂点为g.150971（图3A、B），通过对含断裂点的读长序列比对分析推断另一断裂点位于g.156853附近（图3C）。

**图3:**
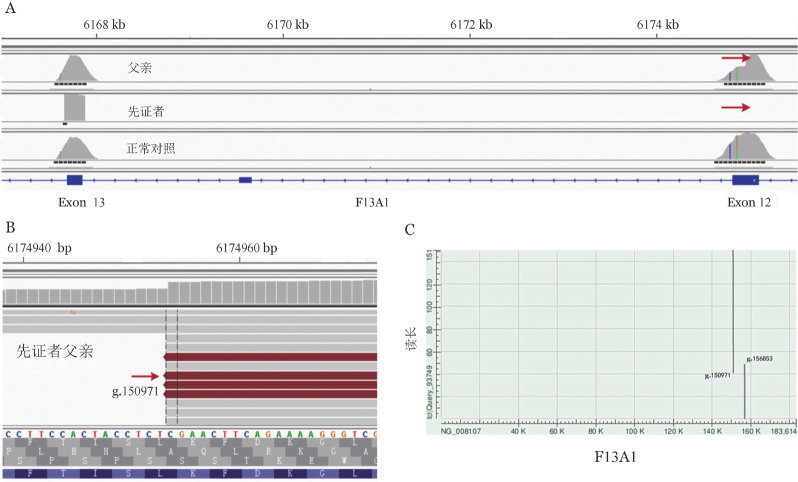
二代测序结果分析图 A 患者父亲、患者和正常对照F13A1基因的exon 12和exon 13二代测序峰图； B 患者父亲近端断裂处定位g.150971； C 含缺失片段的读长序列与F13A1基因比对分析图，断裂区域g.150971-156853

4. PCR扩增及Sanger测序结果：引物F13A1-WT在其他家系成员中扩增出完整的exon 12特异性条带，而先证者此条带缺失（图4A），Sanger测序分析此条带为正常基因型（图4B），说明患者为exon 12纯合缺失。应用引物F13A1-MT扩增可见患者及其父母、兄长1存在突变条带，而患者兄长2未见（图4A），Sanger测序分析该条带为突变基因型，与NG_008107.1序列BLAST比对后提示g.150972-156860之间碱基缺失，共计5 889 bp（图4B），包括F13A1基因的部分12号外显子和内含子（图4C），并且断裂点两端存在8 bp的微小同源序列（图4C）。综合分析两种引物的扩增结果，本研究明确患者为g.150972-156860碱基纯合性缺失，父母及兄长1为携带者，兄长2为正常基因型，遗传家系图见图4D。

**图4:**
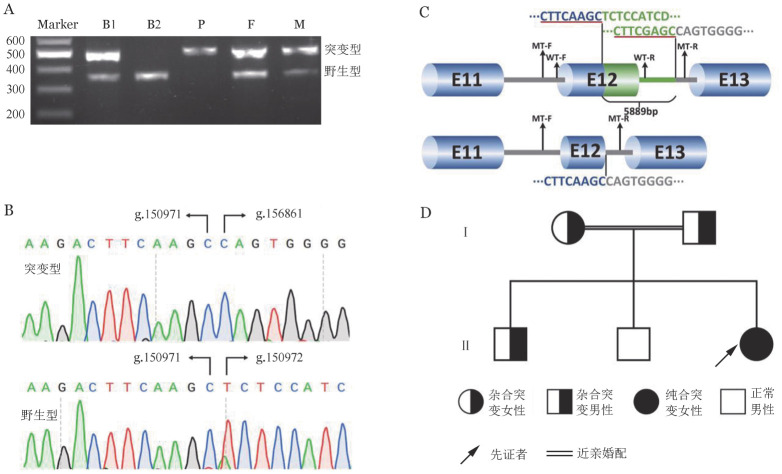
凝血因子XIII缺乏症家系成员F13A1基因突变分析 A 患者家系PCR电泳图（B1：兄长1；B2：兄长2；P：先证者；F：父亲；M：母亲）； B Sanger测序结果； C F13A1基因大片段缺失模式图，红色下划线部分为微同源序列； D 患者家系图

## 讨论

遗传性FXIII缺乏症是一类罕见的遗传性出血性疾病，A亚基（FXIII-A）缺乏症患者的临床症状较重，一般表现为新生儿脐带出血、肌肉血肿、术后及颅内出血，患者致畸致死的概率较高，尚有约1/3患者存在伤口愈合障碍、早期复发性流产等症状[8]。但由于该病发病率极低，并且临床出血表现的异质性大，导致临床认识不足，常规检测不易检出，极易发生漏诊、误诊。随着NGS技术的普及，该类疾病的诊断得到进一步提升。据人类基因突变数据库统计，目前全世界已报告235个遗传性FXIII缺乏症相关突变，其中近90％为F13A1基因突变，主要包括错义/无义突变，小缺失/插入和剪接位点突变，而大片段缺失仅有8例报道，主要通过基因步移和长链PCR技术相结合来检测断裂点位置，该类方法操作复杂，技术上有一定难度[9]–[10]。本研究利用NGS数据鉴定了一个由F13A1基因大片段缺失导致的遗传性FXIII缺乏症家系，为基因大片段缺失的诊断提供参考。

该例患者有明显的出血表型，尿素溶解时间小于30 min，说明血浆FXIII的活性缺失明显，血浆FXIII-A蛋白条带缺失，FXIII-B蛋白条带轻度减低，进一步明确由于血浆FXIII-A缺乏所致。收集患者外周血标本，提取基因组DNA，NGS后采用IGV软件分析发现患者F13A1基因的exon 12信号全部缺失，推测可能存在大片段缺失。其后通过对父亲NGS分析，发现F13A1基因在exon 12出现信号断裂，推断位于exon 12的g.150971位点为断裂点1，其次通过含断裂点的读长序列进行比对分析推断另一断裂点位于g.156853附近。为进一步证实精确的断裂位置，我们设计了扩增缺失突变的引物，扩增涵盖基因F13A1的exon 12和部分intron 12序列，正常片段为6 468 bp，突变片段为579 bp。由于正常片段较大，采用常规PCR扩增体系无法扩增出目的条带，只有含大片段缺失突变能出现579 bp片段，由此根据实验结果判断患者为exon 12纯合性缺失，父母及兄长1为exon 12缺失携带者，兄长2为正常基因型，符合常染色体隐性遗传。对突变片段进行Sanger测序并与NCBI数据库比对分析，证实患者F13A1基因缺失区域在g.150972-156860（NG_008107.1），共缺失5 889 bp。

此外，通过对突变序列分析发现，断裂点两端存在8 bp的微小同源序列（图4C）。在染色体复制或修复过程中微同源介导的非同源末端连接（NHEJ）是导致大片段缺失的常见机制[11]。本病例断裂点两端8 bp微小同源序列存在可能是导致该家系缺失5 889 bp的新连接片段产生机制之一。该大片段缺失后的新的F13A1基因由于intron 12近端剪切位点的丢失可能产生新的F13A1 F剪切体，最终导致无功能的截短蛋白或引起无义突变介导的mRNA降解（NMD）致使患者体内FXIII-A严重缺乏[12]，而本例患者血浆中并未检测到缩短的FXIII-A蛋白，因此推测患者体内由于无义突变介导的mRNA降解致使血浆FXIII-A蛋白缺乏。

综上，本研究报道了1例血浆FXIII-A蛋白严重缺乏的遗传性FXIII缺乏症患者，我们应用NGS技术对一个血浆FXIII-A蛋白严重缺乏的遗传性FXIII缺乏症家系进行基因诊断，明确该病例由F13A1基因大片段缺失所致并发现缺失两端基因8 bp的微同源片段可能是导致大片段缺失的机制之一。该研究是国内首次利用NGS技术鉴定的大片段基因缺失，可为其他遗传性疾病相关基因大片段缺失的诊断提供借鉴。
